# Subanesthetic Isoflurane Reduces Zymosan-Induced Inflammation in Murine Kupffer Cells by Inhibiting ROS-Activated p38 MAPK/NF-*κ*B Signaling

**DOI:** 10.1155/2014/851692

**Published:** 2014-07-23

**Authors:** Hui Wang, Lei Wang, Nan-lin Li, Jun-tang Li, Feng Yu, Ya-li Zhao, Ling Wang, Jun Yi, Ling Wang, Jie-fang Bian, Jiang-hao Chen, Shi-fang Yuan, Ting Wang, Yong-gang Lv, Ning-ning Liu, Xiao-shan Zhu, Rui Ling, Jun Yun

**Affiliations:** ^1^Department of Vascular and Endocrine Surgery, Xijing Hospital, Fourth Military Medical University, No. 15 Changle West Road, Xi'an, Shaanxi 710032, China; ^2^Institute of Anal-Colorectal Surgery, No. 150 Central Hospital of PLA, Luoyang, Henan 451000, China; ^3^State Key Laboratory of Cancer Biology, Department of Immunology, Fourth Military Medical University, Xi'an, Shaanxi 710032, China; ^4^Centre of Material and Drug Supply, No. 150 Central Hospital of PLA, Luoyang, Henan 451000, China; ^5^Department of Anesthesiology, No. 150 Central Hospital of PLA, Luoyang, Henan 451000, China

## Abstract

Volatile anesthetic isoflurane (ISO) has immunomodulatory effects. The fungal component zymosan (ZY) induces inflammation through toll-like receptor 2 or dectin-1 signaling. We investigated the molecular actions of subanesthetic (0.7%) ISO against ZY-induced inflammatory activation in murine Kupffer cells (KCs), which are known as the resident macrophages within the liver. We observed that ISO reduced ZY-induced cyclooxygenase 2 upregulation and prostaglandin E_2_ release, as determined by western blot and radioimmunoassay, respectively. ISO also reduced the production of tumor necrosis factor-*α*, interleukin-1*β*, IL-6, high-mobility group box-1, macrophage inflammatory protein-1*α*, macrophage inflammatory protein-2, and monocyte chemoattractant protein-1 as assessed by enzyme-linked immunosorbent assays. ISO blocked the ZY-induced nuclear translocation and DNA-binding activity of nuclear factor- (NF)-*κ*B p65. Moreover, ISO attenuated ZY-induced p38 mitogen-activated protein kinase (MAPK) activation partly by scavenging reactive oxygen species (ROS); the interregulation that ROS activated p38 MAPK followed by NF-*κ*B activation was crucial for the ZY-induced inflammatory responses in KCs. An *in vivo* study by peritoneal injection of ZY into BALB/C mice confirmed the anti-inflammatory properties of 0.7% ISO against ZY in KCs. These results suggest that ISO ameliorates ZY-induced inflammatory responses in murine KCs by inhibiting the interconnected ROS/p38 MAPK/NF-*κ*B signaling pathways.

## 1. Introduction

Multiple organ dysfunction syndrome (MODS) leads to high morbidity and mortality rates in the intensive care unit and is one of the most urgent public-health challenges worldwide [1, 2]. The liver is frequently the key organ that fails during the development of this syndrome. Kupffer cells (KCs), as the resident macrophages within the liver, are the first to be exposed to the inflammation-triggering materials (bacterial and fungal products as well as other particulate materials) absorbed from the gastrointestinal tract. However, the mechanism of liver injury induced by inflammatory activation of KCs remains to be determined, and the therapeutic regimen requires further investigation.

In 1986, Goris et al. [3] described a zymosan- (ZY-) induced generalized inflammation model by intraperitoneal (IP) injection of ZY in mice, which is recognized as the only model to share numerous characteristics with human MODS, including hepatic injury, and has been adopted by many research groups [4–7]. ZY is a substance derived from the cell wall of the yeast* Saccharomyces cerevisiae*. ZY activates macrophages, which signals through a toll-like receptor 2 (TLR2)/6 heterodimer and subsequently activates the mitogen-activated protein kinase (MAPK) signaling pathway and translocation of NF-*κ*B to the nucleus [8, 9]. MAPK activation may act upstream of NF-*κ*B signaling because the inhibitors of MAPK activation have a negative effect on NF-*κ*B activation [10–13]. NF-*κ*B activation is largely involved in the gene expression of proinflammatory cytokines and chemokines and is responsible for the expression and activity of cyclooxygenase 2 (COX2) [4, 14, 15]. Therefore, the inhibition of downstream ZY signaling may prevent proinflammatory events. Brown et al. [16] demonstrated that the binding of ZY to the principal *β*-glucan receptor (dectin-1) in macrophage modulates cytokine and chemokine production and leads to reactive oxygen species (ROS) generation. ROS can activate MAPKs, inhibitor of *κ*B (I*κ*B), and NF-*κ*B and thus regulates the expression of numerous genes [17]. Moreover, most actual reports claimed that dectin-1, alone or in cooperation with TLR2 as relevant ZY-signaling molecules, initiates inflammation [18].

Isoflurane (ISO) is a widely used inhaled anesthetic, which exerts protective properties mainly through antioxidant and anti-inflammatory activities [19, 20]. ISO exhibits immunomodulatory effects by reducing proinflammatory cytokine [e.g., tumor necrosis factor-*α* (TNF-*α*) and interleukin-1*β* (IL-1*β*)] and chemokine production and by decreasing COX2 expression and prostaglandin E_2_ (PGE_2_) release [6, 7, 14]. Our previous study showed that ISO confers antioxidant activity by scavenging free radicals, including ROS, to reduce oxidative stress-induced lipid peroxidation, thereby resulting in the reduction of inflammatory responses [5]. However, ISO at clinical anesthetic dose (1.2%–2.5%) has adverse effects for critically ill patients, who cannot tolerate its hemodynamic effects, such as vasodilation, myocardial depression, and bradycardia [21]. ISO at less than 1% for sedation weakly interferes with hemodynamics, which is more beneficial for critically ill patients in the intensive care unit [22, 23]. Our recent study demonstrated that ISO at a subanesthetic dose (0.7%) results in reduction of inflammatory responses via antioxidant or anti-inflammatory activity in ZY-induced lung injury in mice [5, 7]. However, the cellular and molecular mechanism of 0.7% ISO against injuries in other organs, such as ZY-induced damage in the liver, remains to be clarified. On the basis of reports on the anti-inflammatory roles of ISO, we investigated the protective molecular mechanisms of 0.7% ISO in ZY-induced inflammatory responses in KCs using* in vitro* and* in vivo* approaches. We examined the anti-inflammatory responses and inhibitory effects of 0.7% ISO on ROS generation and ROS-activated p38 MAPK/NF-*κ*B p65 signaling.

## 2. Materials and Methods

### 2.1. Reagents

All reagents were purchased from Sigma-Aldrich (St. Louis, MO, USA) unless otherwise stated. ISO was obtained from Baxter (Baxter Healthcare Corporation, Deerfield, IL, USA). Rabbit anti-mouse COX2, I*κ*B kinase-*β* (IKK*β*), NF-*κ*B, p38 MAPK, c-Jun N-terminal kinase (JNK), extracellular signal-regulated kinases 1 and 2 (ERK 1/2), *β*-actin, and lamin B antibodies were purchased from Abcam (Cambridge, UK). Rabbit anti-mouse phospho-IKK*β* (pIKK*β*, Ser180), phospho-NF-*κ*B (Ser536), phospho-p38 MAPK (Thr180/Tyr182), phospho-JNK (Thr183/Tyr185), and phospho-ERK1/2 (Thr185/Tyr187) were obtained from Cell Signaling Technology, Inc. (Beverly, MA, USA). Horseradish peroxidase-conjugated anti-rabbit IgG was obtained from Chemicon (Temecula, CA, USA). ROS scavenger (Edaravone, MCI-186) was from Tocris Bioscience (Ellisville, MO, USA). p38 MAPK activation inhibitor (SB202190) and NF-*κ*B activation inhibitor (NAI) were purchased from Biomol (Plymouth Meeting, PA, USA) and Calbiochem (Darmstadt, Germany), respectively. ZY from* S. cerevisiae* was dissolved in isotonic sodium chloride solution to a final concentration of 25 mg/mL. The solution was homogenized by magnetic stirring and sterilized at 100°C for 80 min. All suspensions were freshly prepared prior to use.

### 2.2. Animals and Treatments

Eight-week-old male BALB/C mice (weighing from 22 g to 25 g) were purchased from the Laboratory Animal Center of the Fourth Military Medical University. Animal procedures were approved by the Ethics Committee for Animal Experimentation of the Fourth Military Medical University. All surgeries were performed under anesthesia with sodium pentobarbital, and all efforts were made to minimize suffering. Euthanasia by sodium pentobarbital was consistent with the American Veterinary Medical Association Guidelines on Euthanasia (June 2007).

A MODS model that included liver injury was established by aseptic IP injection of ZY into mice, at a dose of 1 g/kg of body weight, as previously described [5–7]. Sham control (Ctrl) was established by injection of the same volume of normal saline (NS) through the same route.

The animals were placed in a sealed Plexiglas chamber with inflow and outflow outlets. The mice were exposed to ISO via inhalation, in accordance with our previous studies [5–7]. In brief, ISO was delivered into the chamber by air through a tube at a rate of 4 L/min. The ISO flow rate was accurately controlled in real time by regulating the anesthetic vaporizers (Harvard apparatus, USA). The ISO concentration in the outflow hose of the chamber was continuously monitored with a gas analyzer (Brüel & Kjae, Naerum, Denmark) and maintained at 0.7% during the treatment. The oxygen concentration in the chamber was maintained at 21% using supplemental oxygen and was continuously monitored with a gas analyzer (Medical Gas Analyzer LB-2, Model 40 M; Beckman, Fullerton, CA, USA). Carbon dioxide was removed from the chamber gases using Baralyme (Allied Healthcare Products, Inc., St. Louis, MO, USA). The animals without ISO treatment were exposed to room air (RA) in the chamber as the vehicle control. The room and chamber temperatures were maintained from 22°C to 24°C.

*In Vivo Experimental Design. *For* in vivo* studies, 80 mice were randomly assigned to the following groups (*n* = 20 per group). (1) ZY + RA group: mice were given an IP injection of ZY (1 g/kg, dissolved in an NS solution), followed by inhalation of RA (vehicle) for 1 h starting at 1 h and 6 h after ZY administration. (2) ZY + 0.7% ISO group: no differences from the ZY + RA group, except for 1 h inhalation of ISO starting at 1 h and 6 h instead of RA after ZY administration. (3) Ctrl + RA group: no differences from the ZY + RA group, except for administration with NS (Sham Ctrl) instead of ZY. (4) Ctrl + 0.7% ISO group: identical to the Ctrl + RA group, except for 1 h inhalation of ISO starting at 1 h and 6 h instead of RA after NS administration. At the indicated time points after ZY or NS administration, the animals were assessed for ZY-induced liver injury.

*Isolation and Culture of KCs. *The animals were anesthetized using sodium pentobarbital and placed on a plastic tray, to which their limbs were pinned to keep them as straight as possible. A paper pillow was placed under the abdomen to raise the liver above the rest of the organs. The animal was then cut open, and the peritoneal cavity was carefully exposed so as not to damage the liver. Maximum exposure of the peritoneal cavity was ensured to allow full access and visualization and to confirm the location of the portal vein. The thoracic cavity was then opened to expose the heart. A cut was made in the right atrium wall while simultaneously cannulating the portal vein with a 22G catheter connected to a perfusion tube. The liver was perfused with phosphate-buffered saline (PBS) for 4 min at a rate of 10 mL/min until the organ turned pale. Perfusate samples were collected and stored at −80°C until analysis. The liver was then harvested, transferred into a sterile Petri dish, and washed twice with PBS [24].

After Glisson's capsule and connective tissues were removed with scissors, the liver was held with blunt-ended forceps and then thoroughly minced using sharp-ended forceps until the sample was pipettable with a 10 mL pipette. The liver homogenate was transferred to 30 mL of a 1 mg/mL collagenase D solution for digestion. The liver homogenate was stirred at 37°C on a hot plate for 25 min during digestion and then filtered through a cell strainer (100 *μ*m) to remove undigested tissue fragments. The filtrate was transferred into 50 mL Falcon tubes and centrifuged twice at 350 ×g for 8 min at 4°C to remove residual enzymatic solution. The supernatant was discarded, and the pellet was resuspended. Differential centrifugation was performed to separate the parenchymal cells from nonparenchymal cells. After the cell suspension was centrifuged at 50 ×g for 3 min at 4°C, the supernatant was transferred into a fresh 50 mL Falcon tube. Finally, KCs were further isolated and purified from the supernatant using an anti-CD68 MicroBead Kit (Miltenyi Biotec, Germany) in accordance with manufacturer's protocol.

The isolated KCs were cultured and passaged as previously described [24]. Prior to all experiments, More than 99% of the cells were determined viable using Live/Dead violet (Invitrogen, Carlsbad, CA, USA).

*In Vitro Experimental Protocols.* The KCs were seeded on six-well plates, allowed to incubate overnight, and then subjected to ZY (0.5 mg/mL) or control (Ctrl) culture media (CM) treatment for 0.5 h. At 0.5 h after ZY or Ctrl treatment, the media volume in each well was reduced from 2.5 mL to 1 mL for the six-well plates, and the cells were subsequently exposed to RA with or without 0.7% ISO for 0.5 h at 2 L/min in a metabolic chamber (Columbus Instruments, Columbus, OH, USA). During ISO exposure, the ISO concentration (0.7%) was continuously verified by sampling the exhaust gas with a Datex Capnomac (SOMA Technology Inc., Cheshire, CT, USA) [25]. The cells were continuously subjected to ZY or Ctrl treatment for the indicated times. In summary, four treatment groups were established, namely, Ctrl + RA, Ctrl + ISO, ZY + RA, and ZY + ISO. To investigate the inhibitory effects of SB202190, NAI, or MCI-186, KCs were pretreated with or without SB202190 (10 *μ*M), NAI (2 *μ*M), or MCI-186 (50 *μ*M) for 0.5 h, washed out, and treated with ZY (0.5 mg/mL) or new CM for the indicated time periods.

### 2.3. Histological Examination

At 24 h after ZY or NS administration, the livers of mouse euthanized by sodium pentobarbital were harvested and morphological changes were determined. The samples were fixed with 10% formalin for 8 h at room temperature, embedded in paraffin, and then cut into 4 *μ*m thick sections. After deparaffinization and rehydration, the sections were sequentially stained with hematoxylin and eosin. Histologic changes were evaluated by two independent pathologists who had no knowledge of the treatment regimen received by each animal. The degree of liver injury was then subjectively scored from 0 to 3: 0 = absent, 1 = mild, 2 = moderate, and 3 = severe. The scale was used for each of the histologic features, namely, vacuolization, congestion, and necrosis. The final score was obtained by adding the scores of all single evaluations [26].

### 2.4. Quantification of Liver Function and Injury

At 24 h after ZY or NS injection, blood samples were collected and centrifuged (1600 ×g for 3 min at room temperature) to separate the plasma. Alanine aminotransferase (ALT), aspartate aminotransferase (AST), bilirubin, and alkaline phosphatase (ALP) levels were measured by a veterinary clinical laboratory using standard laboratory techniques.

### 2.5. Measurement of PGE_2_ Production

At 24 h after the mice were treated with ZY or NS or the KCs were treated with ZY or CM, PGE_2_ production was quantified from the release of the protein into the liver perfusate or KC culture supernatant. The perfusate samples or culture supernatants were then stored at −80°C until analysis (details are provided in Section 2.4). PGE_2_ was measured in duplicate using radioimmunoassay (RIA) (Amersham, Freiburg, Germany) according to manufacturer's instructions [27].

### 2.6. Measurement of Cytokine and Chemokine Production

At 24 h after the mice were administered with ZY or NS or at 6 h after the KCs were treated with ZY or CM, the cytokine and chemokine levels in the perfusate or KC culture supernatants were measured using commercially available enzyme-linked immunosorbent assay (ELISA) kits. Mouse TNF-*α*, IL-1*β*, IL-6, HMGB-1, MIP-1*α*, MIP-2, and MCP-1 ELISA kits were purchased from R&D Systems (Minneapolis, MN, USA). Optical density was measured on an ELISA plate scanner (CA94089, Molecular Devices, Sunnyvale, Canada). All experiments were performed according to manufacturer's instructions [28].

### 2.7. Western Blot Analysis

At the indicated time points, the cytosolic and nuclear extracts of KCs treated with ZY or CM were obtained using a nuclear extract kit (Active Motif, Carlsbad, CA, USA). All standards and samples were analyzed in triplicate according to manufacturer's instructions [29]. The NF-*κ*B p65 levels were quantified in the nuclear fractions, whereas all other protein levels were quantified in the cytosolic fractions. The ultimate two extracts (cytosolic and nuclear protein) were boiled, separated by sodium dodecyl sulfate polyacrylamide gel electrophoresis, and electrotransferred onto nitrocellulose membranes. Finally, the extracts were immunoblotted with rabbit anti-mouse COX2, IKK*β*, NF-*κ*B, p38 MAPK, JNK, ERK 1/2, phospho-IKK*β* (Ser180), phospho-NF-*κ*B (Ser536), phospho-p38 MAPK (Thr180/Tyr182), phospho-JNK (Thr183/Tyr185), phospho-ERK1/2 (Thr185/Tyr187), *β*-actin, and lamin B antibodies. Equivalent sample loading was confirmed using *β*-actin or lamin B antibodies (Sigma, CA, USA). Detection was performed using an enhanced chemiluminescence assay kit (Pierce, Rockford, IL, USA).

### 2.8. NF-*κ*B DNA-Binding Activity Assay

At 12 h or 24 h after the KCs were treated with ZY/CM* in vitro* or ZY/NS* in vivo*, the NF-*κ*B DNA-binding activity was quantified using a TransAM NF-*κ*B p65 transcription factor assay kit (Active Motif, Carlsbad, CA, USA). Nuclear extracts of the KCs were prepared using a nuclear extraction kit (Active Motif, Carlsbad, CA, USA). All standards and samples were analyzed in duplicate in accordance with manufacturer's instructions [30].

### 2.9. Immunocytochemical Staining

At 12 h or 24 h after the KCs were treated with ZY/CM* in vitro* or ZY/NS* in vivo*, the KCs were fixed with 4% formaldehyde diluted with PBS for 5 min and then washed thrice with PBS. Afterward, the cells were mixed with anti-NF-*κ*B p65 antibodies, applied to the sections, and incubated overnight at 4°C. The next day, the cells were washed thrice with PBS and then incubated with Alexa Fluor 488-labeled secondary antibodies at room temperature for 1 h. Afterward, the cells were washed thrice with PBS and then observed under a fluorescent microscope (BX51, Olympus, Tokyo, Japan). Positive cells in six fields of each culture were quantitated.

### 2.10. Measurement of ROS Production

ROS levels were determined by measuring the oxidative conversion of 2′,7′-dichlorofluorescin diacetate (DCFH-DA) to the fluorescent compound dichlorofluorescin (DCF). In brief, the KCs that were seeded in 96-well plates and underwent various treatments (details in Section 2.5) for the indicated time intervals were incubated with a DCFH-DA solution (15 *μ*M, final concentration) for 0.5 h at 37°C. In addition, the KCs isolated from mice that underwent various treatments (details in Section 2.3) for the indicated time intervals were added to a DCFH-DA solution. DCF fluorescence was determined at 485 nm excitation and 520 nm emission using a fluorescence microplate reader (Safire2, Tecan, Switzerland). All measurements were performed in triplicate.

### 2.11. Statistical Analyses

All values in the figures and texts are expressed as means ± SD of *n* observations. Intergroup differences were determined by Student's two-tailed unpaired *t*-test or one-way ANOVA analysis, followed by Dunnett's post hoc test as appropriate. GraphPad statistical software (GraphPad Software, Inc., San Diego, CA, USA) was used to perform data analysis. In all tests, *P* < 0.05 was considered as statistically significant.

## 3. Results

### 3.1. Subanesthetic ISO Reduces ZY-Induced COX2 Expression and Cytokine and Chemokine Production* In Vitro* in Isolated Murine KCs

**Figure 1:**
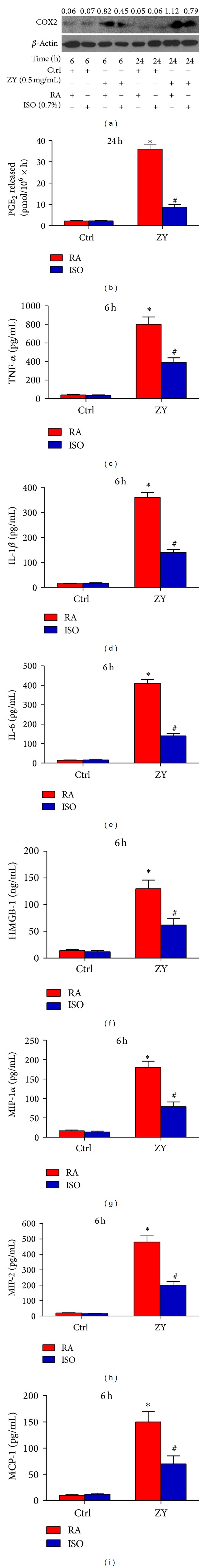
0.7% ISO reduces ZY-induced COX2/PGE_2_ upregulation and cytokine and chemokine production* in vitro*. At 0.5 h after ZY or CM (Ctrl) treatment, the KCs (1 × 10^6^ cells/well in six-well culture plates) were exposed to RA with or without 0.7% ISO for 0.5 h. The cells were continuously stimulated with ZY (0.5 mg/mL) or Ctrl for 6 or 24 h. (a) Western blot was performed to detect the expression of COX2. *β*-Actin was used as the internal control. The ratio form COX2 to *β*-actin is indicated above the bands. (b) RIA was performed to assess PGE_2_ production. ((c)–(i)) ELISA was used to determine the levels of TNF-*α* (c), IL-1*β* (d), IL-6 (e), HMGB-1 (f), MIP-1*α* (g), MIP-2 (h), and MCP-1 (i) in the culture supernatants. Representative data are from three independent experiments and expressed as mean ± SD. *P < 0.05 versus Ctrl + RA or Ctrl + ISO; ^#^ *P* < 0.05 versus ZY + RA. “Time” in the figure represents the time periods of Ctrl or ZY treatment. ZY: zymosan; ISO: isoflurane; Ctrl: control; RA: room air.

Previous studies [31–33] have shown that ZY stimulation induces inflammatory responses, such as COX2/PGE_2_ biosynthesis, and increases the production of proinflammatory cytokines and chemokines in mouse KCs. To investigate the anti-inflammatory effects of 0.7% ISO, we used western blot and RIA to determine COX2 expression and PGE_2_ release. PGE_2_ release is used to evaluate COX2 enzyme activity. We found that posttreatment with 0.7% ISO significantly reduced ZY-upregulated COX2 and PGE_2_ 24 h after ZY treatment (Figures 1(a) and 1(b)). We also measured the levels of cytokines (TNF-*α*, IL-1*β*, IL-6, and HMGB-1) and chemokines (MIP-1*α*, MIP-2, and MCP-1) in the ZY-treated primary KCs using ELISA. We found that ISO posttreatment significantly reduced the ZY-induced upregulation of TNF-*α*, IL-1*β*, IL-6, HMGB-1, MIP-1*α*, MIP-2, and MCP-1* in vitro* (Figures 1(c)–1(i)). These results suggest that 0.7% ISO reduces ZY-induced inflammatory responses in KCs as measured by COX2/PGE_2_ biosynthesis and cytokine and chemokine production.

### 3.2. Subanesthetic ISO Reduces ZY-Induced NF-*κ*B Activation* In Vitro*

**Figure 2:**
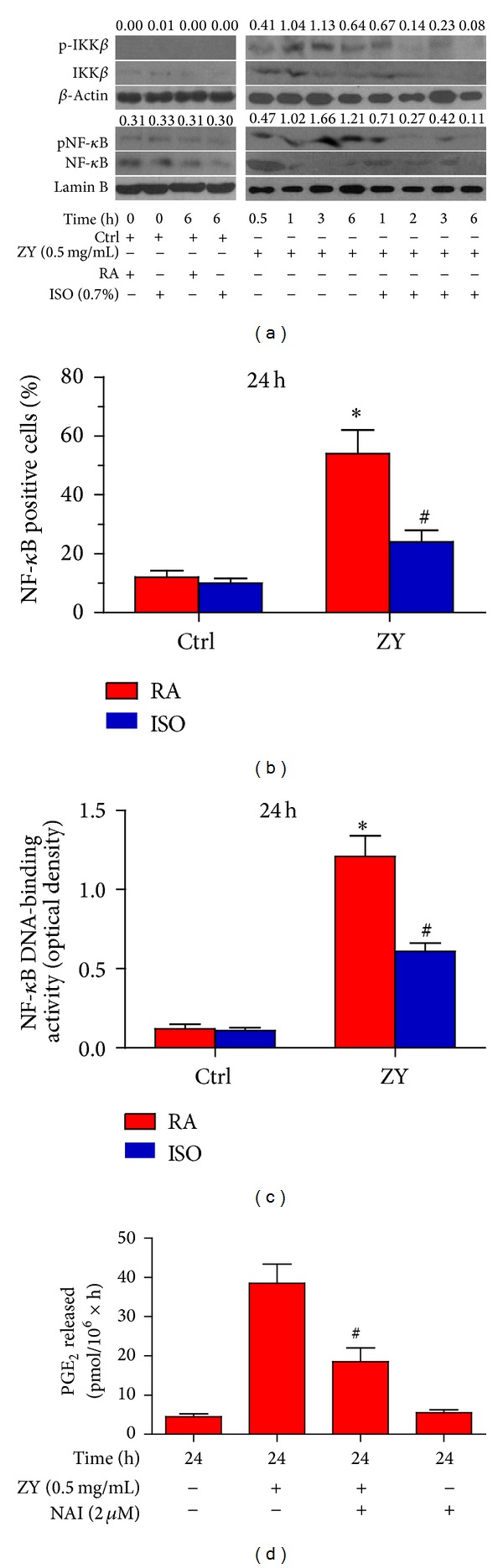
0.7% ISO reduces ZY-induced NF-*κ*B activation* in vitro*. (a) At 0.5 h after ZY or CM (Ctrl) treatment, the KCs (1 × 10^6^ cells/well in six-well culture plates) were exposed to RA with or without 0.7% ISO for 0.5 h. The cells were continuously stimulated with Ctrl for 0 h and 6 h or with ZY (0.5 mg/mL) for 0.5, 1, 2, 3, and 6 h. Western blot was performed to determine the phosphorylation of IKK*β* (Ser180) and NF-*κ*B (Ser536). *β*-Actin and lamin B were used as the internal controls. The ratios from pIKK*β* to IKK*β* and from pNF-*κ*B to NF-*κ*B are indicated above the bands. (b) The KCs were treated as the same way to (a) except that they were continuously stimulated with ZY (0.5 mg/mL) or Ctrl for 24 h. Immunocytochemical staining was performed to assess the nuclear translocation of NF-*κ*B p65 in the KCs immunostained with anti-NF-*κ*B p65 antibody. NF-*κ*B p65-positive cells were then calculated and densitometrically quantified. (c) The KCs were treated as the same way to (b). The NF-*κ*B DNA-binding activity was assayed by determining the optical density. (d) The KCs with or without NAI (2 *μ*M) pretreatment for 0.5 h were treated with ZY (0.5 mg/mL) or Ctrl for 24 h. RIA was performed to detect PGE_2_ production. Representative data are from three independent experiments and expressed as mean ± SD. *P < 0.05 versus Ctrl + RA or Ctrl + ISO; ^#^ *P* < 0.05 versus ZY + RA. “Time” in the figure represents the time periods of Ctrl or ZY treatment. ZY: zymosan; ISO: isoflurane; Ctrl: control; RA: room air.

ISO may act upstream of NF-*κ*B [6, 7], which is an important transcription activator that regulates COX2 and TNF-*α* expression. Utilizing western blot, we found that 0.7% ISO posttreatment reduced the ZY-induced phosphorylation of IKK*β* kinase (Ser180), which is an upstream kinase that serves a critical function in regulating I*κ*B degradation and subsequent NF-*κ*B activation (Figure 2(a)). ISO treatment also reduced ZY-induced phosphorylation of NF-*κ*B (Ser536) after 1 h of ZY challenge (Figure 2(a)). To further investigate the inhibitory effect of ISO on NF-*κ*B signaling in hepatic KCs, we analyzed the nuclear translocation of NF-*κ*B using immunocytochemistry and assessed the DNA-binding activity of NF-*κ*B using TransAM NF-*κ*B transcription factor assay. The results showed that ISO treatment significantly reduced the ZY-induced NF-*κ*B nuclear translocation and DNA-binding activity (Figures 2(b) and 2(c)). To further clarify the role of NF-*κ*B in ZY-induced inflammatory responses of KCs, we pretreated the KCs with NF-*κ*B activation inhibitor NAI. Pretreatment significantly reduced the ZY-induced PGE_2_ upregulation (Figure 2(d)). All these results indicate that 0.7% ISO treatment attenuates ZY-induced inflammatory responses in KCs primarily by reducing NF-*κ*B activation.

### 3.3. Subanesthetic ISO Reduces ZY-Induced p38 MAPK Activation* In Vitro*

**Figure 3:**
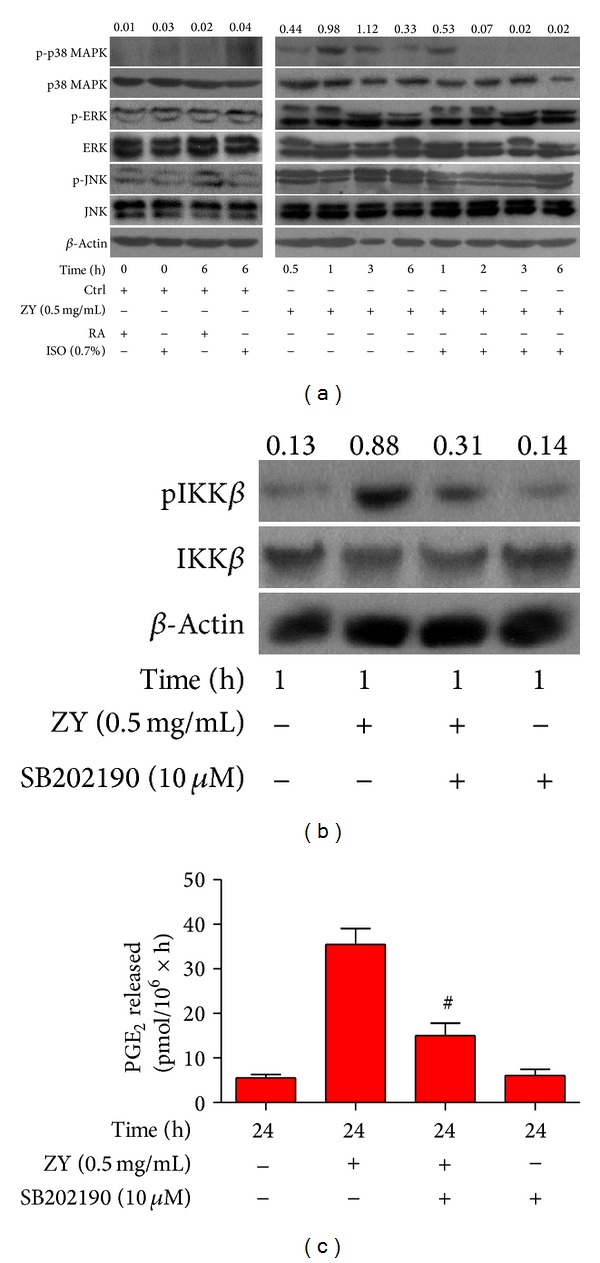
0.7% ISO reduces ZY-induced p38 MAPK activation; p38 MAPK signaling is essential to ZY-induced NF-*κ*B activation and COX2/PGE_2_ generation* in vitro*. (a) At 0.5 h after ZY or CM (Ctrl) treatment, KCs (1 × 10^6^ cells/well in six-well culture plates) were exposed to RA with or without 0.7% ISO for 0.5 h. The cells were continuously stimulated with Ctrl for 0 h and 6 h or ZY (0.5 mg/mL) for 0.5, 1, 2, 3, and 6 h. Western blot analysis was used to assess the phosphorylation of p38 MAPK (Thr180/Tyr182), JNK (Thr183/Tyr185), and ERK1/2 (Thr185/Tyr187). *β*-Actin was used as the internal control. The ratio from p-p38 MAPK to p38 MAPK is indicated above the bands. (b) The KCs with or without SB202190 (10 *μ*M) pretreatment for 0.5 h were treated with ZY (0.5 mg/mL) or Ctrl for 1 h. Western blot was performed to determine the phosphorylation of IKK*β* (Ser180). *β*-Actin was used as the internal control. The ratio from pIKK*β* to IKK*β* is indicated above the bands. (c) The KCs with or without SB202190 (10 *μ*M) pretreatment for 0.5 h were treated with ZY (0.5 mg/mL) or Ctrl for 24 h. RIA was performed to detect PGE_2_ release. Representative data are from three independent experiments and expressed as mean ± SD. *P < 0.05 versus Ctrl + RA or Ctrl + ISO; ^#^ *P* < 0.05 versus ZY + RA. “Time” in the figure represents the time periods of Ctrl or ZY treatment. ZY: zymosan; ISO: isoflurane; Ctrl: control; RA: room air.

MAPK activation can occur upstream of NF-*κ*B signaling [10–13]. We found that 0.7% ISO posttreatment significantly reduced the ZY-induced phosphorylation of p38 MAPK (Thr180/Tyr182) but not those of ERK1/2 (Thr185/Tyr187) or JNK (Thr183/Tyr185) 1 h after ZY treatment (Figure 3(a)). To confirm the regulatory effect of p38 MAPK on NF-*κ*B activation, we demonstrated that pretreatment with SB202190, which is a p38 MAPK activation inhibitor, reduced ZY-induced phosphorylation of IKK*β* (Ser180) 1 h after ZY challenge (Figure 3(b)). We also found that pretreatment with SB202190 significantly reduced ZY-induced upregulation of PGE_2_ in KCs* in vitro* (Figure 3(c)). Overall, these results suggest that 0.7% ISO treatment attenuates ZY-induced inflammatory responses in KCs by reducing p38 MAPK phosphorylation and p38 MAPK-regulated NF-*κ*B activation.

### 3.4. Subanesthetic ISO Reduces ZY-Induced ROS Generation* In Vitro*

**Figure 4:**
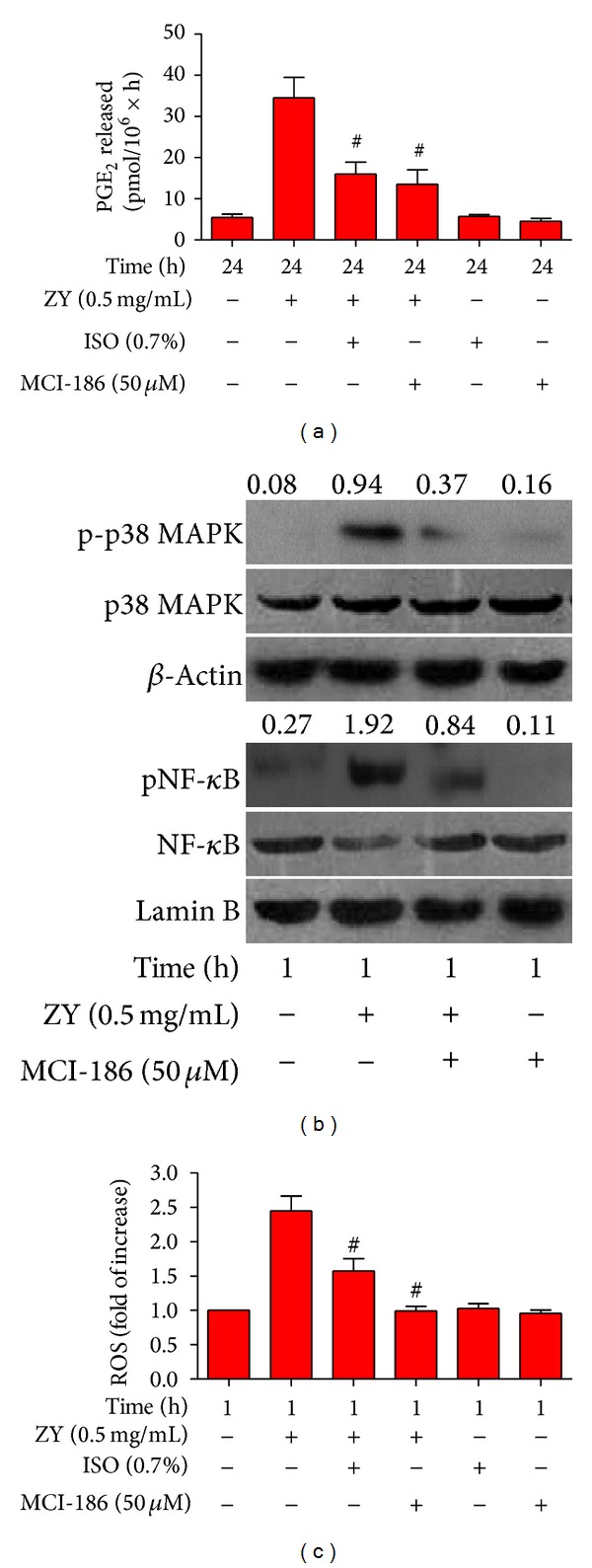
0.7% ISO reduces ZY-induced ROS generation; ROS is essential to ZY-induced activation of p38 MAPK and NF-*κ*B, as well as PGE_2_ production. (a) At 0.5 h after KCs (1 × 10^6^ cells/well in six-well culture plates) were treated with ZY or CM (Ctrl) for 0.5 h with or without MCI-186 (50 *μ*M) pretreatment for 0.5 h, the cells were exposed to RA with or without 0.7% ISO for another 0.5 h. The KCs were continuously treated with ZY (0.5 mg/mL) or Ctrl for 24 h. RIA was performed to detect PGE_2_ production. (b) The KCs with or without MCI-186 (50 *μ*M) pretreatment for 0.5 h were treated with ZY (0.5 mg/mL) or Ctrl for 1 h. Western blot was performed to determine the phosphorylation of p38 MAPK (Thr180/Tyr182) and NF-*κ*B (Ser536). *β*-Actin and lamin B were used as the internal controls. The ratios from p-p38 MAPK to p38 MAPK and from pNF-*κ*B to NF-*κ*B are indicated above the bands. (c) The KCs (5 × 10^4^ cells/well in 96-well culture plates) with or without MCI-186 (50 *μ*M) pretreatment for 0.5 h were stimulated with ZY (0.5 mg/mL) for 1 h with or without 0.7% ISO posttreatment for 0.5 h. DCFH-DA was used to assess the production of intracellular ROS. Representative data are from three independent experiments and expressed as mean ± SD. *P < 0.05 versus Ctrl + RA or Ctrl + ISO; ^#^ *P* < 0.05 versus ZY + RA. “Time” in the figure represents the time periods of Ctrl or ZY treatment. ZY: zymosan; ISO: isoflurane; Ctrl: control; RA: room air.

Our previous study [5] demonstrated that ISO acts as an antioxidant agent to inhibit oxidative stress. ROS is essential in inflammatory promotion because it activates p38 MAPK and downstream NF-*κ*B signaling [17]. Thus, we investigated the effects of 0.7% ISO on ZY-induced ROS signaling. We found that pretreatment with antioxidant MCI-186 significantly reduced the ZY-induced upregulation of PGE_2_ (Figure 4(a)). This result implies the critical role of ROS in ZY-induced inflammatory responses. Western blot results also showed that MCI-186 reduced the ZY-induced phosphorylation of p38 MAPK and NF-*κ*B (Ser536) 1 h after the ZY challenge (Figure 4(b)). Intracellular ROS assay was performed to examine the effect of ISO on ROS. The results demonstrated that ISO significantly reduced the ZY-induced upregulation of ROS* in vitro* (Figure 4(c)). All these data suggest that 0.7% ISO attenuates ZY-induced inflammatory responses in KCs partially by reducing ROS generation and ROS-regulated p38 MAPK and NF-*κ*B activation.

### 3.5. Subanesthetic ISO Reduces ZY-Induced Inflammation* In Vivo* and Ameliorates ZY-Induced Liver Injury

**Figure 5:**
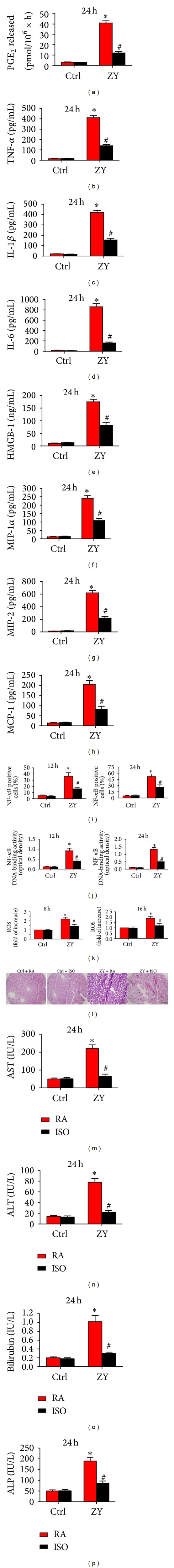
0.7% ISO reduces ZY-induced inflammation* in vivo* and improves liver injury. BALB/C (*n* = 6) mice were treated according to Section 2.3. At 8, 12, 16, or 24 h after injection with ZY or NS (Ctrl), the mice were sacrificed by euthanasia, and theirhepatic KCs and liver perfusates were isolated. ((a)–(h)) The perfusate levels of PGE_2_ (a) and TNF-*α* (b) were determined by RIA, and those of IL-1*β* (c), IL-6 (d), HMGB-1 (e), MIP-1*α* (f), MIP-2 (g), and MCP-1 (h) were determined by ELISA. (i) Fluorescence microscopy was used to determine the nuclear translocation of NF-*κ*B p65 in the KCs immunostained with anti-NF-*κ*B p65 antibody. (j) NF-*κ*B DNA-binding activity of KCs was assayed by determining the optical density. (k) DCFH-DA was used to determine the generation of ROS in the KCs. (l) At 24 h after mice were injected with ZY or Ctrl, the liver tissues from each group were harvested and their morphology was assessed by hematoxylin and eosin staining. Scale bar: 5 *μ*m. At 24 h after mice were administered with ZY or Ctrl, blood samples were collected from all groups and centrifuged to separate plasma. ((m)–(p)) The plasma levels of AST (m), ALT (n), bilirubin (o), and ALP (p) were measured by a veterinary clinical laboratory using standard laboratory techniques. Representative data are from three independent experiments and expressed as mean ± SD. *P < 0.05 versus Ctrl + RA or Ctrl + ISO; ^#^ *P* < 0.05 versus ZY + RA. “Time” in the figure represents the time periods of Ctrl or ZY treatment. ZY: zymosan; ISO: isoflurane; Ctrl: control; RA: room air.

To investigate the anti-inflammatory effects of 0.7% ISO* in vivo*, we analyzed the PGE_2_ release in the perfusate using RIA and detected the levels of cytokines (TNF-*α*, IL-1*β*, IL-6, and HMGB-1) and chemokines (MIP-1*α*, MIP-2, and MCP-1) in the perfusate samples using ELISA. We found that ISO posttreatment significantly reduced the ZY-induced upregulation of PGE_2_, cytokines, and chemokines (Figures 5(a)–5(h)). Western blot results also showed that ISO significantly reduced the ZY-induced phosphorylation of p38 MAPK (Thr180/Tyr182) and NF-*κ*B p65 (Ser536) in the KCs isolated from ZY-treated mice (data not shown). To investigate the effect of ISO on NF-*κ*B signaling in KCs isolated from ZY-challenged mice, we examined the nuclear translocation of NF-*κ*B p65 using immunocytochemistry and assessed the DNA-binding activity of NF-*κ*B p65 using TransAM NF-*κ*B transcription factor assay. We found that ISO posttreatment significantly reduced the ZY-induced nuclear translocation and DNA-binding activity of NF-*κ*B p65 (Figures 5(i)-5(j)). Moreover, intracellular ROS assay results showed that ISO significantly reduced the ZY-induced ROS generation (Figure 5(k)). Histological studies demonstrated that the ZY-challenged mice exhibited significant liver injuries characterized by vacuolization, congestion, and necrosis and ISO treatment ameliorated the inflammatory response and markedly improved the liver architecture (Figure 5(l)). The ZY-treated mice also exhibited significantly higher ALT, AST, bilirubin, and ALP plasma concentrations compared with the control mice (Figures 5(m)–5(p)). These findings were all consistent with the development of liver injury. Posttreatment with ISO significantly reduced the ALT, AST, bilirubin, and ALP plasma concentrations and ameliorated the ZY-induced liver injury (Figures 5(m)–5(p)). All of these results suggest that 0.7% ISO attenuates ZY-induced inflammatory activation in hepatic KCs* in vivo* partly by reducing ZY-induced activation of p38 MAPK and NF-*κ*B p65, restricting ROS generation, thereby ameliorating ZY-induced liver injury.

## 4. Discussion

One of the most critical functions of the liver is the removal of bacterial and fungal products as well as other particulate materials coming from the gut through the portal vein [34]. As the resident macrophages within the liver, KCs are first exposed to the materials absorbed from the gastrointestinal tract [35]. IP injection of the fungal component ZY into mice induces generalized inflammation, which can lead to multiple organ failure, including hepatic injury [4]. ZY is recognized by TLR-2 in immune cells (e.g., KCs) and subsequently causes MAPK and NF-*κ*B activation [8]. MAPK activation can occur as upstream of NF-*κ*B signaling [10–13], as shown by NF-*κ*B downregulation as a result of MAPK signaling attenuation. NF-*κ*B is a critical transcription factor required for the maximal expression of many inflammatory cytokines and chemokines (e.g., TNF-*α*, IL-1*β*, IL-6, PGE2, and MIP-2) involved in the pathogenesis of organ injury [36]. The excessive production of inflammatory mediators such as TNF-*α* and IL-1*β* propagates the extension of inflammation and facilitates systemic inflammation and ultimately contributes to the overall outcome and severity [37]. Additionally, Kinoshita et al. [38] reported that CD11^+^ subset had a more TNF-*α* expression capacity than that of CD68^+^ subset after LPS stimulation. And LPS-stimulated CD68^+^ subset lowly expressed TNF-*α*. However, in this study, we found that zymosan-stimulated CD68^+^ subset produced lots of ROS and simultaneously highly expressed TNF-*α*, which is inconsistent with TNF-*α* expression in LPS-treated CD68^+^ cells. More studies are needed to clarify the contradiction.

Volatile anesthetic ISO has been shown to exhibit anti-inflammatory effects [5, 6]. However, prospects for clinical usage of ISO (1.2%–2.5%) have been hampered due to adverse systemic effects. Our previous report showed that subanesthetic dose of ISO (0.7%) protects against ZY-induced shock by upregulating antioxidant enzymes and reducing inflammatory mediators [5, 7]. We also demonstrated that 0.7% ISO reduces ZY-induced inflammation by particularly targeting NF-*κ*B signaling [7]. However, the molecular mechanisms and signaling pathways underlying the anti-inflammatory actions of 0.7% ISO in ZY-activated macrophages remain undetermined. In this study, we developed* in vitro* and* in vivo* approaches to investigate the ZY signaling-mediated inflammatory responses in KCs. COX2/PGE_2_ biosynthesis and cytokine and chemokine production were used to characterize the responses. The data showed that 0.7% ISO posttreatment significantly reduced the ZY-induced cellular inflammatory responses. ISO treatment also reduced the ZY-activated NF-*κ*B signaling by inhibiting the phosphorylation of IKK*β* (Ser180) and NF-*κ*B (Ser536) and subsequent nuclear translocation of NF-*κ*B. The antioxidant activity of 0.7% ISO, which pharmacologically affects anti-inflammation, has been reported in our previous study [5]. Notably, the present study showed that posttreatment with 0.7% ISO reduced ROS generation and ROS-mediated p38 MAPK activation, which are critical mediators in NF-*κ*B activation. All of these results suggest that ISO posttreatment reduces ZY-induced inflammatory responses in KCs partially by affecting the mechanisms underlying ROS, p38 MAPK, and NF-*κ*B inactivation. Finally, we confirmed that 0.7% ISO posttreatment reduced the ZY-induced increase of ALT, AST, bilirubin, and ALP in plasma and prevented mouse liver dysfunction. We speculated that 0.7% ISO modulated the plasma levels of ALT, AST, bilirubin, and ALP possibly by reducing ZY-induced inflammation in KCs, although this speculation requires verification.

The anti-inflammatory properties of 0.7% ISO (less than 1%) on ZY-activated KCs were demonstrated in this study. However, some reports on mixed actions of clinically relevant doses of ISO (1.2%–2.5%) were found in the literature. Wu et al. [39] reported that a clinically relevant ISO anesthesia (1.4% ISO) promotes Alzheimer's disease neuropathogenesis by increasing the levels of TNF-*α*, IL-1*β*, and IL-6 Kotani et al. [40] demonstrated that 2.1% ISO augments expression of proinflammatory cytokines in rat alveolar macrophages during mechanical ventilation. Li et al. [41] demonstrated that 1.5% ISO-induced cognitive deficits may stem from upregulation of hippocampal IL-1*β*, partially via activation of the canonical NF-*κ*B pathway, in aged rats. However, clinically relevant doses of ISO protect organs (e.g., lung, liver, kidney, and intestine) injury induced by inflammation resulting from endotoxemia or ischemia-reperfusion [42–46]. Moreover, 1.4% ISO administration causes prolonged decreases in agonist-induced superoxide production by neutrophils [47]. These conflicting findings possibly stemmed from different pathological states or molecular mechanisms employed by ISO. Further investigations are needed to clarify the existing contradictions. According to our knowledge, subanesthetic dose of ISO (0.7%) has not been shown to exhibit proinflammatory actions, which exerts protective effects via antioxidant and anti-inflammatory activities.

We appreciate limitations of our studies. First, we should select more time points to measure cytokines/chemokines production and to investigate the more detailed molecular events* in vitro* and* in vivo*. Second, the* in vivo* part of the study could be extended to further confirm the* in vitro* findings, for example, by* in vivo* application of NF-*κ*B inhibitor, p38 MAPK activation inhibitor or ROS scavenger and monitoring of liver damage, and some proinflammatory parameters. Third, we do not investigate whether ZY-induced inflammatory liver injury could be significantly abrogated in COX2−/− mice or following COX2-selective inhibition in COX2+/+ mice. Fourth, we should further investigate whether sodium pentobarbital has effect on ZY-induced KCs activation, and even if the previous study suggested that sodium pentobarbital did not nearly affect inflammatory responses in ZY-challenged mice [5]. Finally, limitations including aging, types of animal, treatment protocol, and the timing periods of administration are critical for evaluating the therapeutic effects of subanesthetic ISO.

The final but most essential aspect in our study is clinical implication of ISO. Unlike most known anti-inflammation agents that induce inhibition of zymosan-evoked peritonitis, such as morphine [48] and TNF-*α*-stimulated gene 6 protein (TSG-6) [49], ISO can permeate cell membranes and successfully target organelles, including the cytosol, mitochondria, and nuclei. In contrast to the clinical doses of ISO preconditioning that have been traditionally utilized, we administered a subanesthetic dose of ISO after the onset of the inflammatory response caused by ZY, and this subanesthetic dose effectively ameliorated the ZY-induced inflammatory responses in KCs* in vitro* and* in vivo* (mice). Importantly, inhaling ISO at a concentration of less than 1% has been used in intensive care unit patients for facilitating mechanical ventilation [50]. Thus, the clinical relevance in our studies of administrating 0.7% ISO after the ZY insult further enhances the appeal of this treatment modality. However, ZY-induced liver injury in mice only partially mimicked the clinical manifestation of human sepsis. Therefore, further investigations are urgently needed to characterize the actions of 0.7% ISO at the cellular and molecular levels. Taken together, we provided evidence that 0.7% ISO exhibited antioxidant and anti-inflammatory activities capable of regulating ROS-mediated p38 MAPK and NF-*κ*B signaling* in vitro* and* in vivo*. The present study of subanesthetic doses of ISO offers a new avenue for future translational and clinical research and holds promise for the development of new therapeutic approaches.

## 5. Conclusions

In the present study, we demonstrated that subanesthetic isoflurane posttreatment significantly reduced zymosan-induced cyclooxygenase 2 upregulation, prostaglandin E_2_ release, and the production of proinflammatory cytokines and chemokines in murine KCs. We especially demonstrated that, for the first time, subanesthetic isoflurane posttreatment ameliorated ZY-induced inflammatory responses in KCs* in vitro* and* in vivo* partially by reducing ROS generation and ROS-regulated p38 MAPK and NF-*κ*B activation.

## Abbreviations

ALP: Alkaline phosphatase
ALT: Alanine aminotransferase
AST: Aspartate aminotransferase
CM: Culture media
COX2: Cyclooxygenase 2
DCF: Dichlorofluorescin
DCFH-DA: 2′, 7′-Dichlorofluorescin diacetate
ELISA: Enzyme-linked immunosorbent assay
HMGB-1: High-mobility group box-1
IL-1*β*: Interleukin-1*β*
IL-6: Interleukin-6
IP: Intraperitoneal
ISO: Isoflurane
KC: Kupffer cell
MAPK: Mitogen-activated protein kinase
MCP-1: Monocyte chemoattractant protein-1
MIP-1*α*: Macrophage inflammatory protein-1*α*
MIP-2: Macrophage inflammatory protein-2
MODS: Multiple organ dysfunction syndrome
NAI: NF-*κ*B activation inhibitor
NF-*κ*B: Nuclear factor-*κ*B
NS: Normal saline
PBS: Phosphate-buffered saline
PGE_2_: Prostaglandin E_2_
RA: Room air
RIA: Radio immunoassay
ROS: Reactive oxygen species
TLR-2: Toll-like receptor-2
TNF-*α*: Tumor necrosis factor-*α*
ZY: Zymosan.
